# Posterior mediastinal extralobar pulmonary sequestration in a neonate with pulmonary artery supply: a case report

**DOI:** 10.3389/fmed.2024.1455978

**Published:** 2024-11-11

**Authors:** Kaiyi Mao, Leibo Wang, Yuchen Mao, Xianhui Shang, Guangxu Zhou, Peng Zhao, Cao Wang, Hong Ma

**Affiliations:** ^1^Department of Pediatric Surgery, Affiliated Hospital of Zunyi Medical University, Zunyi, China; ^2^Department of Pediatric Surgery, Guizhou Children’s Hospital, Zunyi, China; ^3^Urology Surgery, Beijing Jishuitan Hospital Guizhou Hospital, Guiyang, China

**Keywords:** extralobar pulmonary sequestration, neonate, posterior mediastinal mass, misdiagnosis, case report

## Abstract

This paper reports a rare case of extralobar pulmonary sequestration in the posterior mediastinum of a neonate with arterial supply from the pulmonary artery. A 3-day-old male neonate was diagnosed with type II congenital pulmonary airway malformation after prenatal color Doppler ultrasonography showed a lesion with blood supply from the pulmonary artery in the left lung. Post-birth chest computed tomography(CT) showed that the lesion was located in the posterior mediastinum with low density change, mild stripe enhancement after contrast, and no obvious blood supply vessels. A neurogenic tumor was considered for the preoperative diagnosis. The mass was removed by video-assisted thoracoscopic surgery. During the surgery, the mass was observed to be a dark red solid lump with a feeding vessel originating from the pulmonary artery. The postoperative histopathological diagnosis was extralobar pulmonary sequestration. Combined with the preoperative imaging results, it was considered that the nourishing vessels might have intermittent torsion. The patient recovered well after surgery, and no recurrence was observed after 6 months of follow-up. Therefore, the possibility of extralobar pulmonary sequestration cannot be ruled out for posterior mediastinal masses that are not supplied by the descending aorta or without identified feeding vessels.

## Introduction

Pulmonary sequestration (PS) is a rare congenital abnormality involving lung tissue development, accounting for 0.15–6.4% of congenital lung malformations (1). Its main characteristic is the non-functional lung tissue that is separated from the trachea, bronchial tree, and pulmonary artery of normal lung tissue, forming cystic or solid mass structures. Pathologically, PS is classified into two types: intralobar pulmonary sequestration (ILS) and extralobar pulmonary sequestration (ELS), based on whether the abnormal lung tissue shares a pleural envelope with the surrounding lung tissue. ELS constitutes approximately 25% of PS cases and is typically found between the lower left lung and the diaphragm, with occasional occurrences in the mediastinum (2). The blood supply for ELS usually comes from the thoracic aorta or the abdominal aorta, with origins from the pulmonary artery being very rare (3).

Here, we report a unique case of a child whose prenatal ultrasound suggested a mass in the left lung with a blood supply from the pulmonary artery. The lesion progressively enlarged over time. Postnatal enhanced chest CT showed the lesion located in the posterior mediastinum without obvious feeding arteries. During surgery, the lesion appeared dark red and was found to have a feeding artery originating from the pulmonary artery. After complete resection, the pathological results indicated ELS.

## Case presentation

**FIGURE 1:**
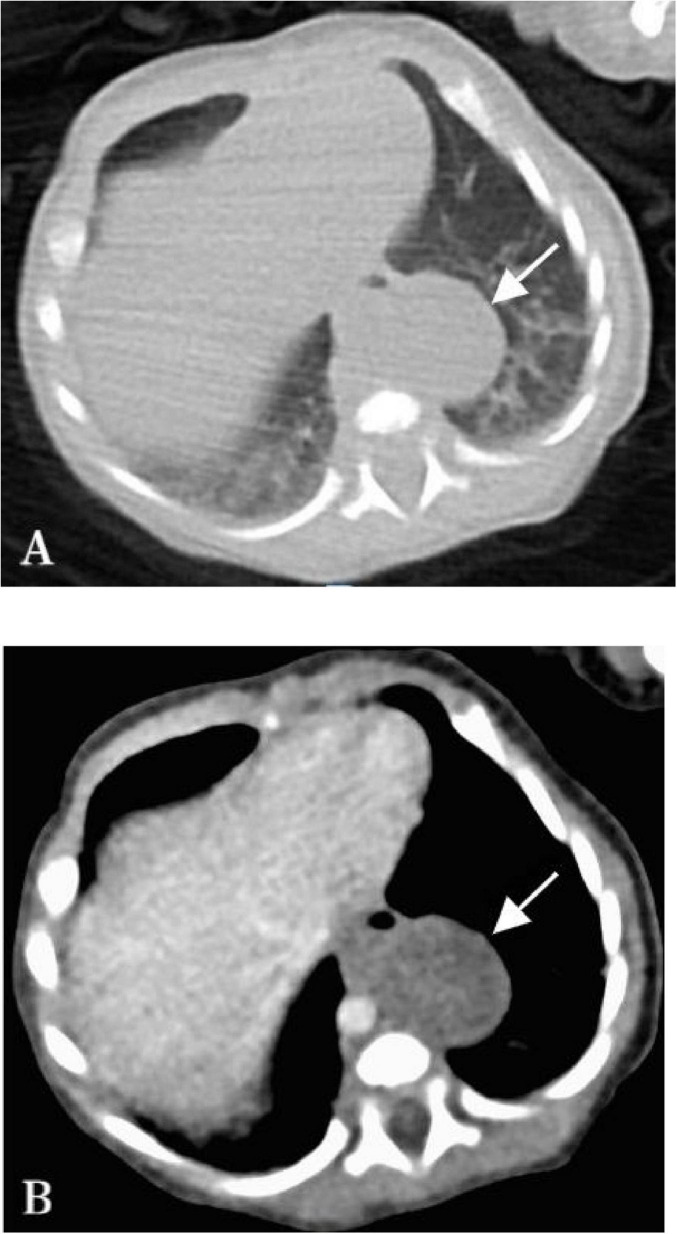
Chest CT image of the child on the 3rd day after birth. **(A)** The plain CT scan shows the presence of an oval-shaped solid mass in the left posterior mediastinum, with no observed cystic structures, measuring approximately 27 × 20 mm with a CT value of 33 HU (arrow). **(B)** Enhanced CT did not reveal significant nourishing vessels within the lesion, mild stripe enhancement was observed within the lesion, which had clear boundaries (arrow).

The male child was admitted to our department 3 days after birth due to prenatal findings of fetal lung abnormalities. During routine prenatal color ultrasound at 24 weeks of gestation, a slightly hyperechoic mass measuring approximately 24 mm × 15 mm × 16 mm was detected in the left thoracic cavity of the fetus, the mass had uniform internal echoes, clear boundaries, and blood supply from the pulmonary artery. Initially, this was suspected to be type II congenital pulmonary airway malformation (CPAM). Subsequent color ultrasound at 30 weeks showed the mass had grown to approximately 30 mm × 26 mm × 20 mm. But the post-birth chest plain CT scan shows the presence of an oval-shaped solid mass in the left posterior mediastinum, with no observed cystic structures, and having a CT value of 33 Hounsfield units (HU) (Figure 1A). After enhancement, no significant nourishing vessels were evident, with only mild strip enhancement observed within the lesion (Figure 1B). Based on these findings, the diagnosis of left type II CPAM was ruled out. The lesion, lacking obvious nourishing vessels and being low-density, located in the posterior mediastinum, initially suggested a neurogenic tumor before surgery. However, prenatal color ultrasound indicated the lesion received blood supply from the pulmonary artery. After consultation within the surgical team, thoracoscopic resection of the posterior mediastinal lesion was planned, focusing on identifying any blood supply artery during the operation.

**FIGURE 2:**
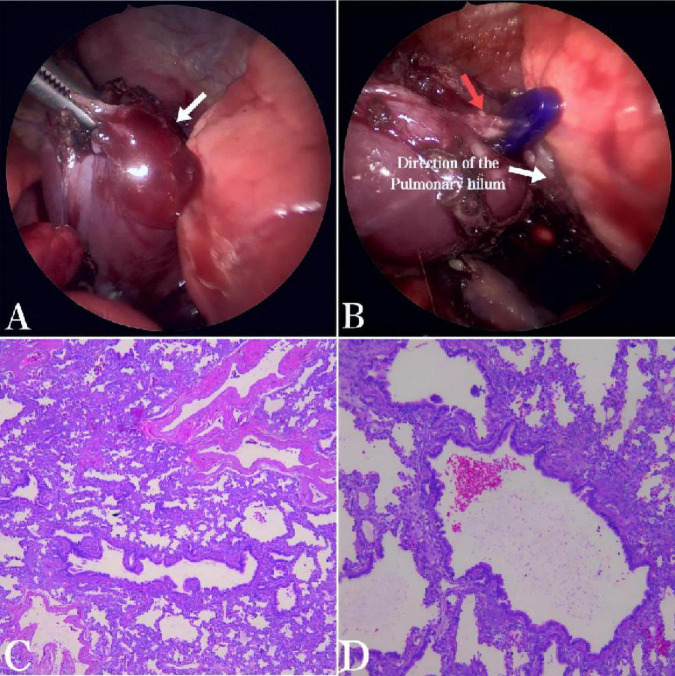
**(A)** During the thoracoscopic examination, a solid mass approximately 30 mm × 25 mm × 10 mm in size, dark red in color, was found in the left posterior mediastinum. The mass was encapsulated by its own visceral pleura, had a smooth surface, was independent of the normal lung tissue, and was not connected to the normal bronchi (arrow). **(B)** A nourishing vessel about 3 mm in diameter was identified at the base of the lesion (red arrow), which was supplied by the pulmonary artery. **(C,D)** Histopathological examination (HE × 50 and HE × 100) showed bronchioles, alveoli, and alveolar structures within the tissue. Bronchiectasis was covered with pseudostratified fibrous columnar epithelium, surrounded by fibromuscular tube walls. Cartilage plates were observed in some bronchial walls, consistent with extralobar pulmonary sequestration.

During the operation, an oval-shaped solid mass, approximately 30 mm × 25 mm × 10 mm in size, was found in the left posterior mediastinum. The mass was encapsulated by its own visceral pleura and was separate from the normal lung tissue, with no connection to the normal bronchi (Figure 2A). Upon careful dissection of the lesion, a nourishing vessel with a diameter of approximately 3 mm was observed at its base, which was supplied by the pulmonary artery (Figure 2B), consistent with the blood supply source indicated by the prenatal ultrasound. Therefore, considered the lesion to be an ELS with atypical blood supply. Complete resection was achieved after clamping the nourishing vessels. Postoperative pathology revealed bronchioles, alveoli, and alveolar structures within the tissue. Bronchiectasis was covered with pseudostratified fibrous columnar epithelium, surrounded by fibromuscular tube walls. Cartilage plates were observed in some bronchial walls, consistent with ELS (Figures 2C,D). The patient recovered well post-surgery, with the thoracic drainage tube removed on the third day and discharge on the fifth day without complications such as pneumothorax, mediastinal emphysema, or atelectasis. Follow-up at 6 months showed the patient’s general condition to be excellent, with no abnormalities detected on chest radiographs.

## Discussion

Some studies indicate that approximately 80% of ELS cases receive arterial blood supply from the thoracic or abdominal aorta, while about 15% are supplied by other systemic arteries, with blood supply from the pulmonary artery being rare (3). In the case presented here, prenatal examination revealed an abnormal left-sided pulmonary lesion supplied by the pulmonary artery. Based on its imaging characteristics and nourishing vessels, prenatal diagnosis primarily considered type II congenital pulmonary airway malformation (CPAM). CPAM is caused by the failure of normal bronchoalveolar structures development, leading to adenomatoid or adenomatous hamartomatous proliferation in the terminal respiratory units without the formation of normal alveoli. Type II CPAM originates in terminal bronchioles, exhibiting smaller cysts and solid areas (4). The key difference between PS and CPAM in prenatal diagnosis lies in their vascular supply. PS derives blood from systemic arteries, often the thoracic aorta, whereas CPAM receives its blood supply from the pulmonary artery. In this case, the prenatal color ultrasound indicated a slightly hyperechoic mass supplied by the left pulmonary artery, making it difficult to distinguish from CPAM, resulting in a misdiagnosis before delivery. Neurogenic tumors are the most common tumors in the posterior mediastinum, whereas ELS is usually located between the left lower lobe of the lung and the diaphragm. Occasionally, it can also occur below the diaphragm, within the diaphragm, but ELS located in the posterior mediastinum is very rare (5). Postnatally, enhanced chest CT in this case indicated a lesion in the posterior mediastinum with low-density changes, slight strip enhancement, and no identifiable nourishing vessels, resembling neurogenic tumor imaging findings. Consequently, CPAM diagnosis was ruled out postnatally, leading to consideration of a neurogenic tumor.

CT examination is highly sensitive for diagnosing ELS, particularly enhanced CT, which can accurately identify the arterial blood supply from systemic circulation. However, studies have demonstrated that in cases where pulmonary sequestration experiences torsion, imaging features on enhanced scans may show non-enhancement or strip enhancement of the mass, without an identifiable abnormal supply artery from systemic circulation (6). In this particular case, enhanced CT after birth revealed minimal enhancement of the tumor with a few strip enhancements and no discernible supplying artery, contradicting prenatal color ultrasound findings. However, during thoracoscopic examination, the lesion appeared dark red with identifiable nourishing vessels. Based on these observations, we hypothesized that the mass may have undergone intermittent torsion, resulting in atypical postnatal imaging findings. Coleman et al. (7) first proposed the phenomenon of intermittent torsion of ELS nourishing arteries. Our case is similar to this phenomenon. In their case, fetal magnetic resonance imaging (MRI) detected the nourishing vessels of ELS. However, a subsequent ultrasound examination revealed no blood flow within the vascular pedicle. Interestingly, during surgery, the ultrasound once again detected the nourishing vessels, indicating possible intermittent torsion. Our case is, therefore, highlighting this phenomenon.

When ELS undergoes torsion necrosis, imaging typically fails to show the supplying arteries, and patients may present with severe abdominal pain, pleural effusion, and other symptoms (8). Some patients may also experience chest pain, dyspnea, and vomiting. Pathological reports often indicate diffuse hemorrhage of the pulmonary parenchyma (9). In our case, however, besides the imaging characteristics, other symptoms were absent. This may be because the nourishing blood vessels of the lesion in this case only underwent intermittent torsion with a relatively mild degree and short duration. Additionally, the child underwent surgery on the fourth day after birth, before the isolated lung tissue could develop ischemic necrosis. Reports suggest that MRI offers advantages in diagnosing ELS with torsion (10, 11). T1-weighted imaging (T1WI) of twisted lung tissue typically shows high signal intensity, indicating lesions combined with bleeding. Furthermore, MRI can visualize cystic or tubular shadows within the mass and accurately delineate the relationship between the lesion, pleura, and normal lung tissue. This capability makes MRI valuable in distinguishing between congenital lung developmental malformations and tumors.

In conclusion, this study presented a rare case of external posterior mediastinal pulmonary sequestration in a neonate with blood supply from the pulmonary artery. Prenatal color ultrasound indicated pulmonary artery supply to the lesion, while postnatal enhanced CT failed to detect nourishing vessels and showed the lesion in the posterior mediastinum, resembling imaging findings seen in neurogenic tumors. These factors collectively contributed to diagnostic challenges and misdiagnosis before surgery. For such cases, relying solely on preoperative CT may not suffice for accurate diagnosis. Preoperative MRI could potentially provide better visualization of abnormal blood vessels and lesion characteristics. Furthermore, meticulous identification of abnormal blood vessels during surgery is crucial. Therefore, even for posterior mediastinal masses that lack blood supply from the descending aorta or have no identifiable nourishing blood vessels, the possibility of ELS should be considered to avoid misdiagnosis.

## Data Availability

The original contributions presented in this study are included in this article/supplementary material, further inquiries can be directed to the corresponding author.
